# Mobility, sleep quality, depression and anxiety as correlates of fatigue in childhood-onset systemic lupus erythematosus

**DOI:** 10.3389/fimmu.2026.1937156

**Published:** 2026-09-16

**Authors:** Mingliang Zheng, Tingting Yan, Yanjuan Zhao, Ting Yu, Zhitian Xiao, Shuli Luo

**Affiliations:** 1 School of Nursing, China Medical University, Shenyang, Liaoning, China; 2 Nursing Department, Shenzhen Children’s Hospital Affiliated to Shantou University Medical College, Shenzhen, Guangdong, China; 3 Department of Rheumatology and Immunology, Shenzhen Children’s Hospital Affiliated to Shantou University Medical College, Shenzhen, Guangdong, China

**Keywords:** anxiety, childhood-onset systemic lupus erythematosus, depression, fatigue, mobility, patient-reported outcomes, sleep quality

## Abstract

**Objective:**

Fatigue is a prevalent and debilitating symptom in childhood-onset systemic lupus erythematosus (cSLE), yet evidence regarding its burden and associated factors remains limited. This study aimed to assess fatigue and identify factors associated with fatigue in patients with cSLE.

**Methods:**

We conducted a cross-sectional study of 167 patients with cSLE recruited from Shenzhen Children’s Hospital between January and December 2025. Fatigue was assessed using the Patient-Reported Outcomes Measurement Information System (PROMIS) Pediatric Fatigue Short Form-10a v2.0. Mobility, anxiety, depression, peer relationships, pain intensity, sleep quality, and clinical characteristics were also assessed. Group differences were examined using the Mann–Whitney U or Kruskal–Wallis H test, associations were evaluated using Spearman rank correlation analysis, and multivariable linear regression was performed to identify factors independently associated with fatigue. A stepwise regression model was additionally conducted as a sensitivity analysis.

**Results:**

The median (IQR) fatigue score was 47.90 (39.00–54.50), and 77 participants (46.1%) experienced at least mild fatigue. Fatigue was positively correlated with depression, anxiety, poorer sleep quality, and pain intensity and negatively correlated with mobility and peer relationships. In the primary multivariable model, lower mobility scores (β = −0.335, P < 0.001), poorer sleep quality (β = 0.261, P < 0.001), higher depression scores (β = 0.230, P = 0.002), and higher anxiety scores (β = 0.155, P = 0.035) were independently associated with greater fatigue. Conventional immunotherapy combined with biologic agents was associated with lower fatigue scores (β = −0.149, P = 0.014), although this variable was not retained in the sensitivity analysis. The primary model explained 64.0% of the variance in fatigue.

**Conclusions:**

Fatigue is common in cSLE and is independently associated with mobility, sleep quality, depression, and anxiety. These findings support a multidimensional view of fatigue in cSLE and highlight the importance of considering physical functioning, sleep quality, and psychological symptoms alongside disease-related factors in comprehensive pediatric rheumatology care.

## 1 Introduction

Systemic lupus erythematosus (SLE) is a chronic multisystem autoimmune disease. Compared with adult-onset SLE, childhood-onset SLE (cSLE) is characterized by higher disease activity and more extensive organ involvement, resulting in a substantially greater overall disease burden (1, 2). Beyond cumulative organ damage and treatment-related toxicity, children and adolescents with cSLE frequently experience persistent symptoms that profoundly impair daily functioning and quality of life (3).

Among these symptoms, fatigue stands out as one of the most prevalent and debilitating in SLE (3, 4). Reported in approximately 58.0% to 70.7% of pediatric patients (5–7), fatigue in cSLE is typically persistent and multifactorial and may not be fully relieved by rest, in contrast to transient fatigue in healthy peers (8).Given that cSLE emerges during a critical period of physical, emotional, and social development, fatigue may have particularly serious consequences, including impaired school attendance, reduced physical activity, emotional distress, and poorer treatment adherence, all of which may contribute to unfavorable long-term outcomes (3, 6, 9).

Despite its clinical importance, evidence on factors associated with fatigue in cSLE remains scarce and inconsistent. Psychological factors, including depression and anxiety, as well as pain, sleep quality, and mobility, have been frequently investigated, but their reported associations with fatigue have varied across studies (6, 9–11). In contrast, disease activity indices, such as the Systemic Lupus Erythematosus Disease Activity Index 2000 (SLEDAI-2000), and treatment regimens have generally shown weak or inconsistent associations with fatigue, suggesting that fatigue is not merely a proxy for disease activity (6, 12, 13). Furthermore, the existing literature has several limitations: most studies have been conducted in Western cohorts with relatively small sample sizes, limiting generalizability to other populations (5, 6, 12, 13); heterogeneous fatigue measures and definitions hamper cross-study comparisons; and many analyses examine isolated domains rather than integrating physical, psychological, and functional dimensions within a unified framework (3, 5–7, 13).

Therefore, the present cross-sectional study aimed to assess fatigue in patients with cSLE and identify its associated factors through multidimensional assessment of physical functioning, psychological status, sleep quality, and clinical characteristics, with the goal of informing clinical evaluation and multidisciplinary management strategies.

## 2 Methods

### 2.1 Study design and setting

This cross-sectional study was designed to assess fatigue and identify factors associated with fatigue in children with cSLE. The study was conducted between January 2025 and December 2025 in the outpatient clinics and inpatient wards of the Department of Rheumatology and Immunology at Shenzhen Children’s Hospital, China.

### 2.2 Participants

Children with cSLE were consecutively recruited from the outpatient clinics and inpatient wards. Eligible participants fulfilled the 2019 European League Against Rheumatism/American College of Rheumatology (EULAR/ACR) classification criteria for systemic lupus erythematosus, were younger than 18 years, and were able to complete the questionnaire assessment with guardian consent (14). Patients were excluded if they had other diagnosed conditions known to independently contribute to fatigue, such as juvenile idiopathic arthritis, hyperthyroidism, heart failure, tuberculosis, malignancy, or fibromyalgia, or if they were participating in other clinical trials that could affect fatigue. A formal power calculation was not performed. Sample size was estimated according to Kendall’s rule for multivariable analysis. Based on 22 candidate variables, a minimum sample of 110–220 participants was required. After allowing for 20% invalid questionnaires or attrition, the target sample size was 133–262 participants. A total of 167 participants were ultimately included in the analysis.

### 2.3 Measures

#### 2.3.1 Demographic and clinical variables

Demographic and clinical data were collected using a researcher-designed questionnaire and medical record review. Demographic variables included age, sex, ethnicity, body mass index (BMI), school status, only-child status, and primary caregiver. Clinical variables included disease activity, disease duration, immunotherapy regimen, vitamin D supplementation, complications, erythrocyte sedimentation rate (ESR), hemoglobin, complement C3, and serum albumin. Disease activity was assessed by a pediatric rheumatologist using the Systemic Lupus Erythematosus Disease Activity Index 2000 (SLEDAI-2000) on the basis of medical records (15). Body mass index was categorized according to age- and sex-specific Chinese criteria for school-age children and adolescents. Immunotherapy regimen was categorized as no immunotherapy, conventional immunotherapy, or conventional immunotherapy combined with biologic agents. Conventional immunotherapy referred to glucocorticoids, hydroxychloroquine, and/or immunosuppressants (16). Detailed glucocorticoid exposure, including current glucocorticoid dose or prednisone-equivalent dose and duration of continuous glucocorticoid therapy, was not collected as an independent study variable.

#### 2.3.2 Fatigue

Fatigue was assessed using the validated Simplified Chinese version of the Patient-Reported Outcomes Measurement Information System (PROMIS) Pediatric Fatigue Short Form-10a v2.0, which assesses fatigue during the previous 7 days (17). The instrument underwent linguistic and cross-cultural adaptation for use in Chinese children and adolescents, and the Chinese version demonstrated good internal consistency, with a Cronbach’s α coefficient of 0.911 (18). The instrument contains 10 items scored on a 5-point Likert scale ranging from 1 (“never”) to 5 (“almost always”). Raw scores were converted to T-scores according to the HealthMeasures scoring manuals (19). Higher T-scores indicate greater fatigue. Fatigue severity was classified as none (<50), mild (50 to <55), moderate (55 to <65), or severe (≥65) (20).

#### 2.3.3 Mobility, anxiety, depression, peer relationships, and pain intensity

Mobility, anxiety, depression, peer relationships, and pain intensity were assessed using the validated Simplified Chinese version of the PROMIS Pediatric Profile-25 (21). The Chinese version has demonstrated good psychometric properties in a pediatric population, with Cronbach’s α coefficients exceeding 0.85 across the multi-item domains. In the present study, Cronbach’s α coefficients were 0.735 for mobility, 0.900 for anxiety, 0.837 for depression, and 0.897 for peer relationships. Except for pain intensity, which is rated on an 11-point numeric rating scale, items are scored on a 5-point Likert scale. Raw scores were converted to T-scores using the HealthMeasures conversion tables (19). For anxiety and depression, higher T-scores indicate more severe symptoms. For mobility, lower T-scores indicate greater functional limitation, whereas for peer relationships, higher T-scores indicate better functioning. The fatigue domain was not administered because it overlapped with the primary outcome measure. In the pain domain, only the pain intensity item was used.

#### 2.3.4 Sleep quality

Sleep quality was assessed using the validated Chinese version of the Pittsburgh Sleep Quality Index (PSQI) (22), which evaluates sleep quality during the previous month across seven components. The Chinese version has demonstrated satisfactory reliability and validity. In addition, psychometric evaluation of a Chinese-language PSQI in children and adolescents aged 6–18 years demonstrated acceptable internal consistency (Cronbach’s α = 0.71), excellent test–retest reliability (intraclass correlation coefficient = 0.90), and evidence of construct and convergent validity. Total scores range from 0 to 21, with higher scores indicating poorer sleep quality (22).

#### 2.3.5 Data collection

Questionnaires were administered by trained researchers during outpatient visits or on the day of hospital admission. Before data collection, the study purpose and procedures were explained using standardized instructions, and written informed consent was obtained from the participants and their legal guardians in accordance with institutional requirements. Disease-related data were extracted from the medical record system. Participants aged 8 years or older completed the questionnaires themselves, whereas parent-proxy versions were used for children younger than 8 years. When participants had difficulty understanding questionnaire items, trained researchers provided standardized explanations without influencing their responses. Completed questionnaires were checked on site for missing responses, and all data were independently entered by two researchers using Microsoft Excel.

#### 2.3.6 Statistical analysis

Statistical analyses were performed using IBM SPSS Statistics version 27.0 (IBM Corp., Armonk, NY, USA). Normality was assessed using the Kolmogorov–Smirnov test together with quantile–quantile plots. Continuous variables were summarized as mean ± standard deviation (SD) or median with interquartile range (IQR), as appropriate, and categorical variables were summarized as frequencies and percentages. Group differences in fatigue scores were examined using the Mann–Whitney U test or Kruskal–Wallis H test, as appropriate. Associations between fatigue and mobility, anxiety, depression, peer relationships, pain intensity, and sleep quality were evaluated using Spearman rank correlation analysis.

Multivariable linear regression was performed with fatigue score as the dependent variable. Variables that were statistically significant in the univariable or correlation analyses and considered clinically relevant were entered simultaneously into the multivariable model using the enter method. Immunotherapy regimen was dummy coded, with no immunotherapy as the reference category. Multicollinearity was assessed using variance inflation factors (VIFs), with values >5 considered indicative of problematic multicollinearity. Model assumptions were evaluated by inspection of residual plots and normal probability plots. A stepwise multivariable linear regression model was additionally performed as a sensitivity analysis to examine the robustness of the primary findings. All statistical tests were two-sided, and P < 0.05 was considered statistically significant.

## 3 Results

### 3.1 Participant characteristics

A total of 173 questionnaires were distributed, of which 167 were valid and included in the analysis, yielding a valid response rate of 96.5%. The mean age of the participants was 12.91 ± 2.57 years (range, 6–17 years), and 85.0% were female. The median (IQR) disease duration was 17 (1–36) months, and the median (IQR) SLEDAI-2000 score was 8 (4–10). Approximately half of the participants (50.3%) were receiving conventional immunotherapy combined with biologic agents. Other demographic and disease-related characteristics are summarized in **Table 1**.

**Table 1:** Demographic and disease-related characteristics of participants.

Variable	Category	n (%) or value
Age, years	—	12.91 ± 2.57
Age group, years	6–11	48 (28.7)
	12–17	119 (71.3)
Sex	Male	25 (15.0)
	Female	142 (85.0)
Ethnicity	Han	151 (90.4)
	Minority	16 (9.6)
Body mass index	Moderate/severe underweight	9 (5.4)
	Mild underweight	6 (3.6)
	Normal	136 (81.4)
	Overweight	13 (7.8)
	Obesity	3 (1.8)
School status	Not attending school	13 (7.8)
	Primary school or below	61 (36.5)
	Junior high school	63 (37.7)
	Senior high school or above	30 (18.0)
Primary caregiver	Father	47 (28.1)
	Mother	108 (64.7)
	Grandparent	12 (7.2)
Only child	Yes	28 (16.8)
	No	139 (83.2)
Disease duration, months	—	17 (1–36)
Disease duration category	Newly diagnosed	55 (32.9)
	Previously diagnosed	112 (67.1)
SLEDAI-2000 score	—	8 (4–10)
Disease activity	No or minimal	58 (34.7)
	Mild	55 (32.9)
	Moderate	29 (17.4)
	Severe	25 (15.0)
Lupus nephritis	Yes	80 (47.9)
	No	87 (52.1)
Immunotherapy regimen	No immunotherapy	42 (25.1)
	Conventional immunotherapy	41 (24.6)
	Conventional immunotherapy combined with biologic agents	84 (50.3)
Vitamin D supplementation	No or irregular supplementation	59 (35.3)
	400 IU/day	58 (34.7)
	800 IU/day	50 (29.9)
Erythrocyte sedimentation rate, mm/h	—	9 (4–24)
Hemoglobin, g/L	—	120 (108–130)
Complement C3, g/L	—	0.88 (0.72–1.05)
Serum albumin, g/L	—	42.30 (39.40–44.20)

Data are presented as n (%), mean ± standard deviation (SD), or median (interquartile range [IQR]), as appropriate. Newly diagnosed was defined as disease duration <3 months at the time of survey. Disease activity was classified as no or minimal (0–4), mild (5–9), moderate (10–14), or severe (≥15). Conventional immunotherapy referred to glucocorticoids, hydroxychloroquine, and/or immunosuppressants. SLEDAI-2000, Systemic Lupus Erythematosus Disease Activity Index 2000; IU, international units.

### 3.2 Fatigue status and related patient-reported outcomes

The median (IQR) fatigue score was 47.90 (39.00–54.50). Overall, 77 participants (46.1%) experienced at least mild fatigue, including 37 (22.2%) with mild fatigue, 33 (19.8%) with moderate fatigue, and 7 (4.2%) with severe fatigue. The median (IQR) scores for the other patient-reported outcomes were 45.50 (41.20–57.10) for mobility, 49.90 (35.60–54.80) for anxiety, 46.80 (37.70–54.60) for depression, 46.70 (42.60–51.90) for peer relationships, 1 (0–3) for pain intensity, and 4 (2–6) for sleep quality. Detailed distributions of fatigue and the other patient-reported outcomes are presented in **Table 2**.

**Table 2:** Fatigue and other patient-reported outcomes.

Variable	Category	n (%) or value
Fatigue score	—	47.90 (39.00–54.50)
Fatigue severity	None	90 (53.9)
	Mild	37 (22.2)
	Moderate	33 (19.8)
	Severe	7 (4.2)
Mobility	—	45.50 (41.20–57.10)
	Normal	104 (62.3)
	Mild limitation	33 (19.8)
	Moderate limitation	24 (14.4)
	Severe limitation	6 (3.6)
Anxiety	—	49.90 (35.60–54.80)
	None	100 (59.9)
	Mild	26 (15.6)
	Moderate	36 (21.6)
	Severe	5 (3.0)
Depression	—	46.80 (37.70–54.60)
	None	101 (60.5)
	Mild	39 (23.4)
	Moderate	25 (15.0)
	Severe	2 (1.2)
Peer relationships	—	46.70 (42.60–51.90)
	Excellent	30 (18.0)
	Good	104 (62.3)
	Fair	27 (16.2)
	Poor	6 (3.6)
Pain intensity	—	1 (0–3)
	None	94 (56.3)
	Mild	61 (36.5)
	Moderate	10 (6.0)
	Severe	2 (1.2)
Sleep quality	—	4 (2–6)
	Good	112 (67.1)
	Mild impairment	49 (29.3)
	Moderate impairment	5 (3.0)
	Severe impairment	1 (0.6)

Data are presented as n (%) or median (interquartile range [IQR]). Scores for fatigue, mobility, anxiety, depression, and peer relationships are presented as T-scores. Higher T-scores indicate greater fatigue, anxiety, and depression; lower mobility T-scores indicate greater functional limitation; and higher peer-relationship T-scores indicate better functioning. Pain intensity was rated on an 11-point numeric rating scale, and sleep quality was assessed using the Pittsburgh Sleep Quality Index.

### 3.3 Univariable analysis of fatigue

Among the demographic and disease-related variables examined, fatigue scores differed significantly across immunotherapy regimens (P = 0.002), with the lowest median fatigue score observed among participants receiving conventional immunotherapy combined with biologic agents. No significant differences in fatigue scores were observed according to age group, sex, ethnicity, only-child status, body mass index category, school status, primary caregiver, disease activity, disease duration, lupus nephritis status, or vitamin D supplementation (**Table 3**).

**Table 3:** Univariable analysis of fatigue according to demographic and disease-related variables.

Variable	Category	Fatigue score, median (IQR)	Z/H	P value
Age group, years	6–11	46.05 (34.95–53.50)	−1.772	0.076
	12–17	50.02 (39.00–56.60)		
Sex	Male	45.40 (35.60–54.50)	−1.011	0.312
	Female	48.50 (39.00–54.78)		
Ethnicity	Han	47.90 (39.00–53.50)	−0.934	0.350
	Minority	51.85 (39.95–58.60)		
Only child	Yes	50.02 (45.50–56.60)	−1.569	0.117
	No	46.70 (36.90–53.50)		
Body mass index category	Moderate/severe underweight	53.50 (41.15–61.60)	8.480	0.075
	Mild underweight	46.05 (41.63–57.60)		
	Normal	46.70 (39.00–53.50)		
	Overweight	51.30 (36.90–58.10)		
	Obesity	65.60 (60.05–65.60)		
School status	Not attending school	54.50 (33.60–64.60)	3.809	0.283
	Primary school or below	46.70 (36.90–53.50)		
	Junior high school	47.90 (40.90–56.60)		
	Senior high school or above	49.60 (37.83–55.28)		
Primary caregiver	Father	49.10 (39.00–54.50)	0.017	0.991
	Mother	46.70 (39.00–54.37)		
	Grandparent	49.05 (34.95–58.60)		
Disease activity	No or minimal	46.70 (36.25–53.50)	1.508	0.680
	Mild	50.20 (39.00–54.00)		
	Moderate	46.70 (36.90–57.10)		
	Severe	47.90 (39.00–59.60)		
Disease duration category	Newly diagnosed	49.10 (39.00–55.60)	−0.979	0.327
	Previously diagnosed	46.70 (39.00–53.88)		
Lupus nephritis status	Yes	46.70 (39.00–53.88)	−0.677	0.498
	No	50.20 (39.00–55.60)		
Immunotherapy regimen	No immunotherapy	52.40 (46.70–57.60)	12.459	0.002
	Conventional immunotherapy	51.30 (37.95–57.10)		
	Conventional immunotherapy combined with biologic agents	45.40 (34.30–52.40)		
Vitamin D supplementation	No or irregular supplementation	49.10 (40.90–55.60)	2.198	0.138
	400 IU/day	49.10 (40.90–55.85)		
	800 IU/day	43.95 (34.30–53.50)		

Data are presented as median (interquartile range [IQR]). Z values are reported for Mann–Whitney U tests, and H values are reported for Kruskal–Wallis H tests. Newly diagnosed was defined as disease duration <3 months at the time of survey. Conventional immunotherapy referred to glucocorticoids, hydroxychloroquine, and/or immunosuppressants.

### 3.4 Correlation analysis

Fatigue scores were negatively correlated with mobility and peer relationships and positively correlated with anxiety, depression, pain intensity, and sleep quality (all P < 0.05). The strongest correlations were observed for mobility (r = −0.661), depression (r = 0.638), sleep quality (r = 0.622), and anxiety (r = 0.619) (**Table 4**).

**Table 4:** Correlation analysis of fatigue and related variables.

Variable	r	P value
Mobility	−0.661	<0.001
Anxiety	0.619	<0.001
Depression	0.638	<0.001
Peer relationships	−0.259	0.009
Pain intensity	0.340	<0.001
Sleep quality	0.622	<0.001

r values are Spearman rank correlation coefficients. All tests were two-sided.

### 3.5 Multivariable analysis of factors associated with fatigue

Variables that were statistically significant in the univariable or correlation analyses and considered clinically relevant were entered into the multivariable linear regression model. No problematic multicollinearity was detected (VIFs, 1.125–2.351; tolerance values, 0.425–0.888), and inspection of the residual plots indicated that the model assumptions were adequately met. Lower mobility scores (β = −0.335, P < 0.001), poorer sleep quality (β = 0.261, P < 0.001), higher depression scores (β = 0.230, P = 0.002), and higher anxiety scores (β = 0.155, P = 0.035) were independently associated with greater fatigue. In addition, receipt of conventional immunotherapy combined with biologic agents was independently associated with lower fatigue scores (β = −0.149, P = 0.014). Peer relationships and pain intensity were not independently associated with fatigue after adjustment. The model explained 64.0% of the variance in fatigue scores (R = 0.800, R² = 0.640, adjusted R² = 0.622; F = 35.173, P < 0.001) (**Table 5**).

**Table 5:** Multivariable linear regression analysis of factors associated with fatigue.

Variable	B	SE	β	t	P value
Constant	41.139	7.330	—	5.613	<0.001
Conventional immunotherapy	−2.166	1.444	−0.089	−1.500	0.136
Conventional immunotherapy combined with biologic agents	−3.140	1.264	−0.149	−2.485	0.014
Mobility	−0.441	0.078	−0.335	−5.656	<0.001
Anxiety	0.163	0.077	0.155	2.126	0.035
Depression	0.304	0.097	0.230	3.148	0.002
Peer relationships	0.061	0.058	0.053	1.044	0.298
Sleep quality	0.895	0.216	0.261	4.148	<0.001
Pain intensity	−0.037	0.270	−0.007	−0.136	0.892

B, unstandardized regression coefficient; SE, standard error; β, standardized regression coefficient. Immunotherapy regimen was dummy coded with no immunotherapy as the reference category. Model fit: R = 0.800; R² = 0.640; adjusted R² = 0.622; F = 35.173; P < 0.001.

### 3.6 Sensitivity analysis

A stepwise multivariable linear regression model was additionally performed as a sensitivity analysis. The final model retained mobility, sleep quality, depression, and anxiety, with regression coefficients in the same directions as those observed in the primary model. Conventional immunotherapy combined with biologic agents was not retained in the stepwise model. Overall, the associations of mobility, sleep quality, depression, and anxiety with fatigue remained consistent across the two modeling approaches, supporting the robustness of these core findings (**Table 6**).

**Table 6:** Sensitivity analysis using stepwise multivariable linear regression.

Variable	B	SE	β	t	P value
Constant	41.972	6.298	—	6.664	<0.001
Mobility	−0.448	0.077	−0.341	−5.853	<0.001
Anxiety	0.155	0.076	0.148	2.026	0.044
Depression	0.317	0.095	0.240	3.343	0.001
Sleep quality	0.886	0.210	0.258	4.220	<0.001

B, unstandardized regression coefficient; SE, standard error; β, standardized regression coefficient. Model fit: R = 0.790; R² = 0.624; adjusted R² = 0.615; F = 67.313; P < 0.001.

## 4 Discussion

In this cross-sectional study of 167 patients with cSLE, nearly half of the participants experienced at least mild fatigue. Greater fatigue was independently associated with lower mobility scores, poorer sleep quality, and higher levels of depression and anxiety. Conventional immunotherapy combined with biologic agents was also associated with lower fatigue scores in the primary multivariable model, although this association was not retained in the sensitivity analysis. Taken together, these findings support a multidimensional view of fatigue in cSLE, involving physical functioning, sleep, and psychological factors in addition to disease-related characteristics.

The fatigue burden observed in this cohort was clinically meaningful. The proportion of participants with at least mild fatigue (46.1%) was somewhat lower than that reported in some previous cSLE studies (58.0%–70.7%), although comparisons across studies should be made cautiously. Differences in fatigue estimates may reflect variation in assessment instruments, recall periods, thresholds used to define fatigue, and the clinical characteristics of study populations (6, 7, 12). In the present study, the PROMIS Pediatric Fatigue Short Form and T-score-based severity categories provided a standardized approach to grading fatigue severity, which may partly account for differences from studies using symptom checklists or other fatigue measures (6, 7). Cultural differences in symptom perception and reporting may also contribute, although this possibility requires further investigation. Nevertheless, our findings are consistent with previous pediatric lupus research indicating that fatigue is common and clinically relevant and that fatigue and mood-related symptoms are closely related to impaired health-related quality of life in youth with cSLE (5, 6).

Among the factors identified, mobility showed the strongest standardized association with fatigue (β = −0.335, P < 0.001). The PROMIS mobility domain reflects patients’ perceived ability to perform everyday physical activities, such as walking, climbing stairs, and participating in usual activities, rather than maximal exercise performance under testing conditions. This finding is broadly consistent with previous cSLE research showing that poorer self-reported physical functioning is associated with greater fatigue (13). By contrast, earlier exercise-based studies using objective fitness indicators, such as peak oxygen uptake and ventilatory anaerobic threshold, did not identify significant associations with fatigue (12). These differences may reflect the distinct constructs captured by perceived mobility and physiological exercise capacity. Fatigue in cSLE may therefore be particularly relevant to limitations in day-to-day functional participation. Clinically, fatigue assessment should be accompanied by evaluation of mobility and daily functioning, with attention to maintaining functional participation in school, home, and other routine activities.

Poor sleep quality was another factor independently associated with greater fatigue (β = 0.261, P < 0.001). This finding is consistent with recent pediatric evidence showing that sleep disturbances, including poorer sleep efficiency, irregular sleep patterns, and greater wake after sleep onset, are associated with higher fatigue levels in youth with cSLE (9). Adult SLE studies have similarly shown that sleep disturbances frequently coexist with fatigue, anxiety, and depression (23). Importantly, however, the PSQI assesses sleep quality over the preceding month, whereas the PROMIS Pediatric Fatigue Short Form assesses fatigue over the preceding 7 days. This difference in recall periods may have reduced the temporal comparability of the two measures and should therefore be considered when interpreting the observed association. In addition, the cross-sectional design does not permit determination of the temporal direction of the relationship between sleep quality and fatigue. Nevertheless, the observed association supports the clinical relevance of evaluating sleep quality, continuity, and regularity when assessing fatigue in patients with cSLE.

Depressive and anxiety symptoms also emerged as important correlates of fatigue. Both depression (β = 0.230, P = 0.002) and anxiety (β = 0.155, P = 0.035) remained independently associated with fatigue in the multivariable model, with depression showing a somewhat stronger standardized association. This pattern is consistent with previous cSLE literature showing that fatigue and depressive symptoms are closely related to poorer health-related quality of life (5, 6), as well as with more recent evidence indicating that depression and anxiety are common in cSLE and adversely affect quality of life and family functioning (10). More broadly, qualitative and review-based literature has highlighted the substantial psychological and social burden associated with cSLE and the need for holistic care (3). These findings reinforce the view that fatigue should not be interpreted solely as a physical symptom or a surrogate for inflammatory disease activity. Rather, fatigue appears to coexist with a broader cluster of psychological, sleep-related, and functional difficulties. Routine assessment of depressive and anxiety symptoms may therefore help clinicians better characterize fatigue complaints and identify patients who may benefit from further psychological assessment or support.

Several variables commonly considered relevant to fatigue were not independently associated with fatigue in the present study. Disease activity, disease duration, lupus nephritis status, and most demographic characteristics were not significantly associated with fatigue in the univariable analyses. This finding is broadly consistent with earlier cSLE studies suggesting that fatigue does not necessarily parallel standard indicators of disease activity or other routine clinical characteristics (12, 13). In addition, although pain intensity and peer relationships were associated with fatigue in the correlation analyses (r = 0.340 and r = −0.259, respectively; both P < 0.05), neither remained independently associated with fatigue after multivariable adjustment. This attenuation does not imply that pain or peer-related functioning is clinically unimportant; rather, their associations with fatigue may overlap with those of mobility, sleep quality, depression, and anxiety. This interpretation is consistent with broader cSLE literature showing that children’s everyday experiences are shaped by interactions among symptoms, functional limitations, treatment demands, and social participation (3). Overall, these findings support a multidimensional approach to fatigue assessment rather than attribution of fatigue to any single clinical or psychosocial factor.

Receipt of conventional immunotherapy combined with biologic agents was associated with lower fatigue scores in the primary multivariable model (β = −0.149, P = 0.014); however, this finding requires cautious interpretation. Importantly, the treatment variable was not retained in the stepwise sensitivity analysis, indicating that this association was less robust than those observed for mobility, sleep quality, depression, and anxiety. Moreover, treatment exposure was categorized at the regimen level, and the analysis could not account for specific biologic agents, current glucocorticoid dose, duration of continuous glucocorticoid therapy, cumulative glucocorticoid exposure, or treatment response. Evidence from adult SLE suggests that biologic therapy may be accompanied by improvements in patient-reported outcomes, including fatigue (24, 25), but the present cross-sectional findings cannot establish a treatment effect. Confounding by indication and differences in treatment response may also have influenced the observed association. Accordingly, the association between biologic-containing regimens and fatigue should be regarded as hypothesis-generating rather than confirmatory. Prospective studies with more detailed treatment data, including specific biologic agents, current glucocorticoid dose, duration of glucocorticoid therapy, and cumulative glucocorticoid exposure, are needed to clarify the relationship between treatment exposure and fatigue.

Several limitations should be acknowledged. First, this was a single-center study based on convenience sampling, which may limit the generalizability of the findings to other populations and healthcare settings. Second, the cross-sectional design precludes conclusions regarding the temporal or causal relationships between fatigue and its associated factors. Third, most variables were assessed using self-report or parent-proxy measures, which may have introduced reporting bias and shared-method variance. In addition, sleep quality was assessed over the preceding month, whereas fatigue was assessed over the preceding 7 days. This mismatch in recall periods may have reduced the temporal comparability of the two measures and should be considered when interpreting their observed association. Fourth, potentially relevant factors, including objective physical activity, objective sleep parameters, detailed medication exposure, treatment-related adverse effects, and family or school contextual factors, were not fully captured. In particular, current glucocorticoid dose and duration of continuous glucocorticoid therapy were not collected as independent study variables and therefore could not be included as covariates in the multivariable analysis. Consequently, residual confounding related to glucocorticoid exposure cannot be excluded. Future multicenter longitudinal studies incorporating detailed treatment data together with objective measures of activity and sleep are needed to clarify the relationships between fatigue and physical, psychological, and clinical factors. Despite these limitations, this study extends the existing evidence by providing data from a relatively large Chinese pediatric cohort, a population that remains underrepresented in cSLE fatigue research (3, 5, 7, 12, 13). Moreover, mobility, sleep quality, anxiety, depression, peer relationships, pain intensity, and clinical characteristics were examined within a single analytical framework, allowing a more integrated evaluation of factors associated with fatigue.

Overall, the present findings support routine fatigue assessment in pediatric rheumatology care and suggest that fatigue management in cSLE may benefit from multidisciplinary attention to physical functioning, sleep quality, depression, and anxiety in addition to disease control. Prospective studies are needed to determine whether interventions targeting these potentially modifiable factors can reduce fatigue and improve health-related quality of life in patients with cSLE.

## 5 Conclusions

Fatigue is common in childhood-onset systemic lupus erythematosus and is independently associated with mobility, sleep quality, depression, and anxiety. These findings support routine fatigue assessment as part of comprehensive pediatric rheumatology care and highlight the importance of considering physical functioning, sleep quality, and psychological symptoms when evaluating fatigue in patients with cSLE. Multidisciplinary management addressing these domains may help improve the overall assessment and management of fatigue, although longitudinal and interventional studies are needed to determine whether modifying these factors can reduce fatigue burden.

## Data Availability

The original contributions presented in the study are included in the article/supplementary material. Further inquiries can be directed to the corresponding author/s.
